# Ex-vivo and live animal models are equally effective training for the management of a penetrating cardiac injury

**DOI:** 10.1186/s13017-016-0104-3

**Published:** 2016-08-31

**Authors:** Yoshimitsu Izawa, Shuji Hishikawa, Tomohiro Muronoi, Keisuke Yamashita, Hiroyuki Maruyama, Masayuki Suzukawa, Alan Kawarai Lefor

**Affiliations:** 1 Center of Development for Advanced Medical Technology, Jichi Medical University, 3311-1 Yakushiji, Shimotsukeshi, Tochigiken 329-0498 Japan; 2 Department of Emergency and Critical Care Medicine, Jichi Medical University, 3311-1 Yakushiji, Shimotsukeshi, Tochigiken 329-0498 Japan; 3 Department of Digestive Surgery, Jichi Medical University, 3311-1 Yakushiji, Shimotsukeshi, Tochigiken 329-0498 Japan

**Keywords:** Trauma surgery, Ex-vivo training, Surgical education, Simulation, Objective Structured Assessment of Technical Skills, Self-efficacy

## Abstract

**Background:**

Live tissue models are considered the most useful simulation for training in the management for hemostasis of penetrating injuries. However, these models are expensive, with limited opportunities for repetitive training. Ex-vivo models using tissue and a fluid pump are less expensive, allow repetitive training and respect ethical principles in animal research. The purpose of this study is to objectively evaluate the effectiveness of ex-vivo training with a pump, compared to live animal model training. Staff surgeons and residents were divided into live tissue training and ex-vivo training groups. Training in the management of a penetrating cardiac injury was conducted for each group, separately. One week later, all participants were formally evaluated in the management of a penetrating cardiac injury in a live animal.

**Results:**

There are no differences between the two groups regarding average years of experience or previous trauma surgery experience. All participants achieved hemostasis, with no difference between the two groups in the Global Rating Scale score (ex-vivo: 25.2 ± 6.3, live: 24.7 ± 6.3, *p* = 0.646), blood loss (1.6 ± 0.7, 2.0 ± 0.6, *p* = 0.051), checklist score (3.7 ± 0.6, 3.6 ± 0.9, *p* = 0.189), or time required for repair (101 s ± 31, 107 s ± 15, *p* = 0.163), except overall evaluation (3.8 ± 0.9, 3.4 ± 0.9, *p* = 0.037). The internal consistency reliability and inter-rater reliability in the Global Rating Scale were excellent (0.966 and 0.953 / 0.719 and 0.784, respectively), and for the checklist were moderate (0.570 and 0.636 / 0.651 and 0.607, respectively). The validity is rated good for both the Global Rating Scale (Residents: 21.7 ± 5.6, Staff: 28.9 ± 4.7, *p* = 0.000) and checklist (Residents: 3.4 ± 0.9, Staff Surgeons: 3.9 ± 0.3, *p* = 0.003). The results of self-assessment questionnaires were similarly high (4.2–4.9) with scores in self-efficacy increased after training (pre: 1.7 ± 0.8, post: 3.2 ± 1.0, *p* = 0.000 in ex-vivo, pre: 1.9 ± 1.0, post: 3.7 ± 0.7, *p* = 0.000 in live). Scores comparing pre-training and post-evaluation (pre: 1.7 ± 0.8, post: 3.7 ± 0.9, *p* = 0.000 in ex-vivo, pre: 1.9 ± 1.0, post: 3.8 ± 0.7, *p* = 0.000 in live) were increased.

**Conclusion:**

Training with an ex-vivo model and live tissue training are similar for the management of a penetrating cardiac injury, with increased self-efficacy of participants in both groups. The ex-vivo model is useful to learn hemostatic skills in trauma surgery.

**Electronic supplementary material:**

The online version of this article (doi:10.1186/s13017-016-0104-3) contains supplementary material, which is available to authorized users.

## Background

Simulation training is essential for learning a variety of skills in medical training. Concerns for patient safety and super-specialization have increased the need for simulation-based training. Simulation training for hemostasis in trauma surgery is especially desired because hemostasis is critical to save severely injured patients and there are limited opportunities for surgeons to learn the skills needed to treat traumatically injured patients in clinical practice [1, 2]. The number of surgical procedures performed by trainees has been decreasing with advancements in non-operative management [1]. Various simulation courses are conducted around the world in part to make up for limited real-life experience [3–5].

Live tissue training is considered by some to be the most useful simulation for training in the hemostasis of traumatic injuries [6, 7]. However, these programs are expensive, repetitive training is impractical, and ethical issues regarding the use of live animals have gained intense focus [7]. These factors limit the conduct of live tissue training programs. Other methods, such as cadaver training, virtual reality and bench model training, are less suitable for training in hemostatic skills because of decreased reality or a total lack of bleeding [6, 7].

Ex-vivo tissue can be used for simulation training for technical skills [8, 9]. It is generally considered to have mid-level fidelity, is financially reasonable and conducive to repetitive training. We previously conducted a pilot study in the use of a circulation pump for ex-vivo training in trauma surgery, with subjective evaluation [8]. The pump mimics circulation, and appears similar to live tissue. However, objective assessment of this method has not been conducted.

The purpose of this study is to objectively evaluate the effectiveness of ex-vivo training with a pump, compared to live tissue training, for the management of a penetrating cardiac injury.

## Methods

Institutional Animal Experiment Committee approval was obtained before beginning this study. This study was conducted in the Center for Development of Advanced Medical Technology (CDAMtec) at Jichi Medical University.

### Participants

Staff surgeons and residents at Jichi Medical University Hospital took part in this study. This program was the first time any of the participants managed a penetrating cardiac injury. Participants were surveyed regarding their level of training, years of experience and details of their previous trauma surgery experience.

### Study design

**Fig. 1:**
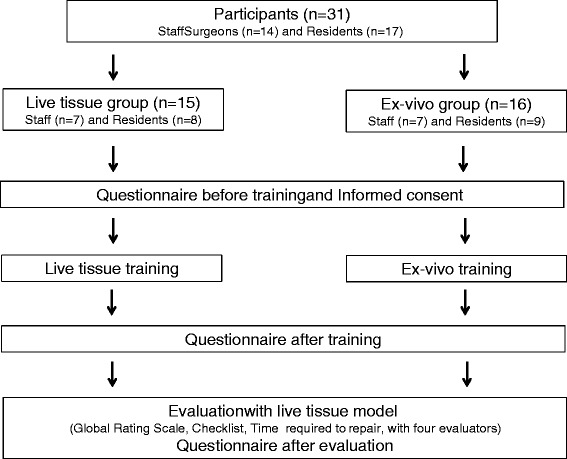
Study flowchart

**Fig. 2:**
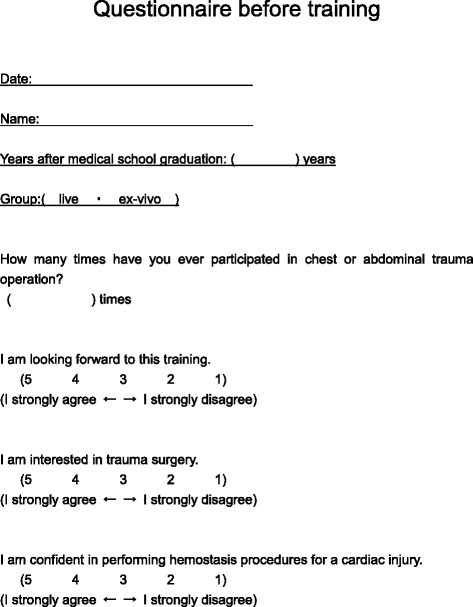
Pre-training questionnaire

**Fig. 3:**
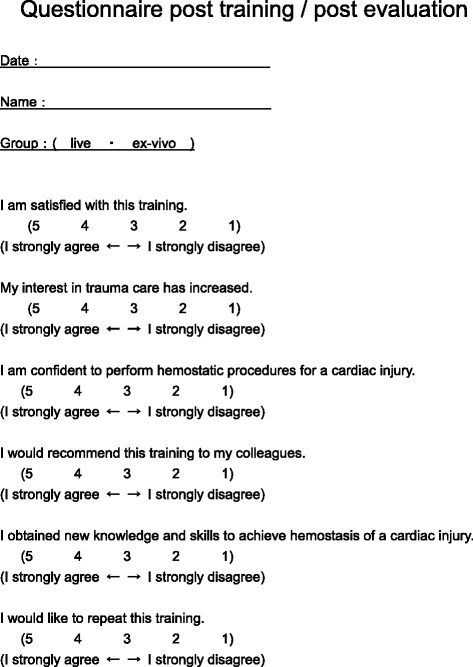
Post-training and post-evaluation questionnaire

This is a comparative study comparing ex-vivo training and live tissue training (Fig. 1). Participants were divided into two groups, an ex-vivo training group and a live tissue training group. Participants were randomly assigned based on their last name. However, there were a few changes because of emergency clinical responsibilities on the day of the training. After completing a pre-training questionnaire (Fig. 2) and obtaining informed consent about participation in the study, participants were trained by experienced trauma surgeons to achieve hemostasis of a penetrating cardiac wound and then repair the wound with explanations and demonstrations. Participants then practiced achieving hemostasis of a one centimeter cardiac injury with help from the instructor. The ex-vivo group did this using an ex-vivo tissue model, and the live group performed this on a live, anesthetized pig. After the training, a post-training questionnaire was completed (Fig. 3).

One week after the training session, all participants managed a standard one centimeter penetrating cardiac wound in a live animal using the skills acquired during the previous training session. Each participant managed the wound individually, and was assessed with an Objective Structured Assessment of Technical Skills (OSATS) instrument. After the evaluation, a post-evaluation survey form was completed by the participants (Fig. 3).

### Ex-vivo training session (Fig. 4)

Ex-vivo training was performed using porcine hearts from animals used in other experiments or training. The ex-vivo hearts were harvested and frozen, the frozen organs thawed and connected to a circulation pump before the training session. During the training, a one centimeter penetrating wound was made in the right ventricle. Participants learned how to stop bleeding initially with digital pressure, and then repair the injury with direct suture, under the guidance of an instructor.

**Fig. 4:**
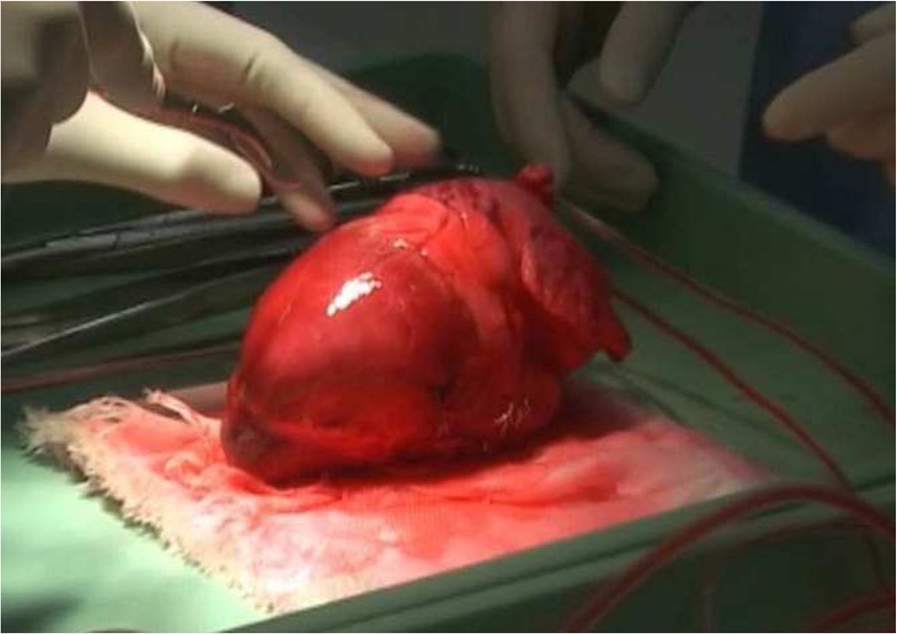
Ex-vivo tissue: cardiac injury

### Live tissue training session (Fig. 5)

Live tissue training was performed with pigs (Crossbred KCG or Mexican hairless miniature pig, approximately 30–40 kg under general anesthesia (Sevoflurane 2–5 %). After performing a median sternotomy and incising the pericardium, a penetrating injury about one centimeter long was made in the right ventricle by the instructor. Participants achieved hemostasis using digital pressure and then repaired the injury with direct suture.

**Fig. 5:**
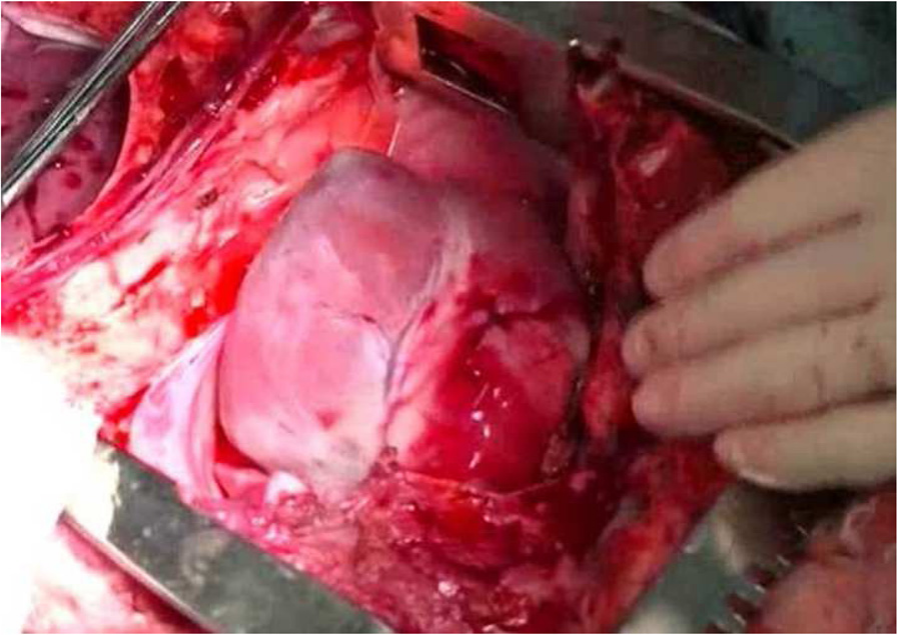
Live tissue training: cardiac injury

### Evaluation session

Participants were evaluated by direct observation in their management of a cardiac injury in the live animal model conducted one week after the training session. The animals were the same type and size used in the training session. The evaluation session was conducted similarly for all participants. Four evaluators assessed each participant, and completed an evaluation form using the OSATS instrument. The time required to repair the injury by each of the participants was recorded. The evaluators were blinded to whether participants had undergone training with live tissue or an ex-vivo model.

There were six evaluators in total, and four of the six assessed the performance of each participant using the OSATS. Two of the six were experienced surgeons with more than 25y experience in acute care surgery. Two were board certified surgeons and others included an experienced veterinarian, and an experienced veterinary technician with more than 30 years of experience in veterinary care and surgery.

**Fig. 6:**
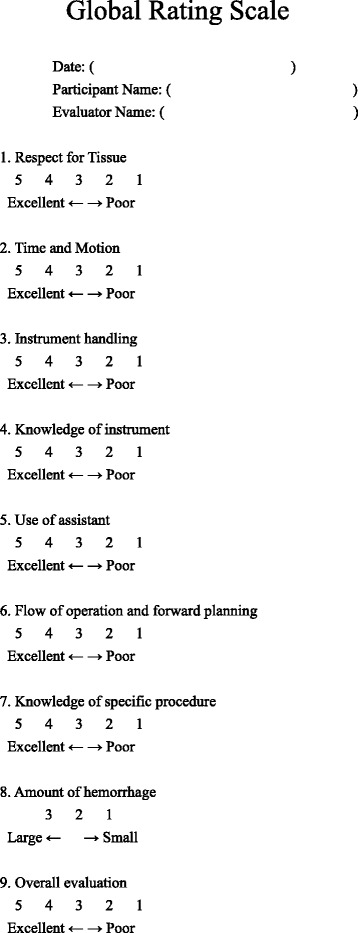
Global Rating Scale score sheet, including evaluations of amount of hemorrhage and overall evaluation

**Fig. 7:**
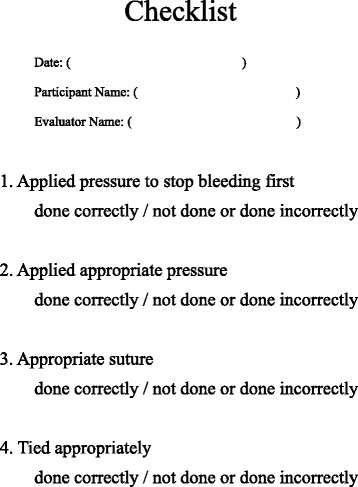
Evaluation checklist

OSATS is widely accepted as a reliable and valid evaluative tool for simulation [10, 11]. This OSATS instrument included a Global Rating Scale and checklist. The Global Rating Scale includes nine items (Fig. 6). Item 1–7 are standard, and we added two items including, “amount of hemorrhage” and “overall evaluation”. In eight of the nine items, a five-point Likert scale (1 = poor ~ 5 = excellent) was used. One item, “amount of hemorrhage”, was a three-point qualitative scale (1 = little, 2 = intermediate and 3 = a lot). The checklist included four Yes/No questions (Fig. 7). The questions on the checklist are task-specific and more concrete, different from the global rating scale.

Reliability and validity of the OSATS were evaluated in this study. The internal consistency reliability was analyzed by Cronbach’s α. The inter-rater reliability was analyzed by inter-class correlation. The validity was evaluated by comparing the outcome of the Global Rating Scale (as the sum of the seven component scores, each score range 1–5, overall range 7–35) and checklist score (4 items scored 0 or 1, overall range 0–4) of staff surgeons and residents, using a sum of the scores.

Participants completed questionnaires at three separate time points: pre-training post-training and post-evaluation. The questionnaires utilized a five point Likert scale (1 = poor ~ 5 = excellent) and included an assessment of self-efficacy (self-confidence) (Figs. 2 and 3).

### Statistical analysis

Data were analyzed with SPSS (IBM, Armonk NY) version 22.0. Continuous variables are presented as means with standard deviations. Mann Whitney’s *U* test is used for continuous variables because most data were non-normal distributions. Wilcoxon signed-rank test is used for paired continuous variables. Inter-class correlation is used for the assessment of inter-rater reliability. Statistical significance was set at a *P*-value less than .05.

## Results

### Study participants

There are no statistical differences between the two study groups, although the number of years after medical school graduation and trauma surgery experience in the ex-vivo training group was slightly higher than in the live tissue group (Table 1).

**Table 1:** Characteristics of study participants: years after medical school graduation and previous experience in trauma surgery

Study group (*n* = number of participants)	Ex-vivo	Live tissue	*P*-value
	(*n* = 16; residents 9, Staff Surgeons 7)	(*n* = 15; residents 8, Staff Surgeons 7)	
Years after medical school graduation (mean years (Standard Deviation))	4.6 (4.9)	3.5 (2.4)	0.654
Prior experience in chest and abdominal trauma surgery (mean number of procedures (Standard Deviation))	4.5 (7.0)	2.2 (3.1)	0.861

### Evaluation of management of a penetrating cardiac injury

All participants were able to achieve hemostasis. Table 2 shows the results of the Global Rating Scale, amount of hemorrhage, overall evaluation, checklist and time required to repair. There was a significant difference in the overall evaluation between the two study groups, with the ex-vivo group being significantly higher. The amount of hemorrhage before achieving hemostasis was higher in the live tissue trained group, but this difference did not quite reach statistical significance.

**Table 2:** Global Rating Scale score, amount of hemorrhage, overall evaluation, checklist and time required to repair comparing ex-vivo and live tissue groups

Study group (*n* = number of evaluation forms)	Ex-vivo (*n* = 64)	Live tissue (*n* = 60)	*P* value
Global Rating Scale (sum of scores for seven items, Range 7–35)	25.2 (6.3)	24.7 (6.3)	0.646
Amount of hemorrhage (Range 1–3)	1.6 (0.7)	2.0 (0.6)	0.051
Overall evaluation	3.8 (0.9)	3.4 (0.9)	0.037
Checklist	3.7 (0.6)	3.6 (0.9)	0.189
Time required (seconds)^a^	101 (31), n=16	107 (15), n=15	0.163

Scores shown are mean (Standard Deviation), using a 5-point Likert scale (range 1–5), except hemorrhage which is rated on a 3-point scale (1 = little, 2 = intermediate, 3 = a lot). Checklist scores are range 0–4. Scores shown are the mean of the four evaluations for all participants

^a^values of n shown for time are number of participants

Table 3 shows the internal consistency reliability and inter-rater reliability for both the Global Rating Scale and checklist. The internal consistency reliability scores were better in both groups than the inter-rater reliability, but the results were reasonable for all four evaluations. Although two instructors could not participate in all evaluations, two different instructors served as substitutes, and the inter-rater reliability scores remained excellent.

**Table 3:** Internal consistency reliability and inter-rater reliability of Global Rating Scale and checklist scores

Study group		Ex-vivo	Live tissue
Internal consistency reliability, Cronbach’s α (Seven items)	Global Rating Scale	0.966	0.953
	Checklist	0.570	0.636
Inter-rater reliability, inter-class correlation score (Four evaluators)	Global Rating Scale	0.719	0.784
	Checklist	0.651	0.607

Table 4 shows the validity by comparing the Global Rating Scale and checklist scores between residents and staff surgeons. There is a significant difference between residents and staff surgeons, with the validity for both rating scales significantly greater for staff surgeons than for residents.

**Table 4:** Validity of Global Rating Scale and checklist scores: Residents and staff surgeons, four evaluations for each participant (Mann–Whitney *U* test)

Group (*n* = number of evaluation forms)	Residents (*n* = 68)	Staff surgeons (*n* = 56)	*P* value
Global Rating Scale (mean and standard deviation, sum of seven scores each score range 1–5, with overall range 7–35)	21.7 (5.6)	28.9 (4.7)	0.000
Checklist score (range 0–4)	3.4 (0.9)	3.9 (0.3)	0.003

Tables 5 and 6 show the results of pre-training, post-training and post-evaluation questionnaires. Table 5 shows that the two study groups had no differences in their subjective self-assessment before the training. Table 6 shows no differences in subjective evaluations comparing the two study groups after training and after the evaluation session. There was a small, but not statistically significant difference in the satisfaction scores after the training session, but this difference did not appear after the evaluation session.

**Table 5:** Pre-training questionnaire

Study group (*n* = number of participants)	Ex-vivo (*n* = 16)	Live tissue (*n* = 15)	*P* value
I am looking forward to this training.	4.4 (0.7)	4.4 (0.8)	0.984
I am interested in trauma surgery.	4.2 (0.9)	4.2 (0.9)	0.984
I am confident in performing hemostatic procedures for a cardiac injury.	1.7 (0.8)	1.9 (1.0)	0.545

Scores shown are mean (Standard Deviation), using a 5-point Likert scale (range 1–5)

**Table 6:** Post training and post evaluation questionnaires

Study group (*n* = number of participants)	Questionnaire	Ex-vivo (*n* = 16)	Live tissue (*n* = 15)	*P* value
I am satisfied with this training.	Post-training	4.5 (0.6)	4.9 (0.3)	0.078
	Post-evaluation	4.8 (0.4)	4.9 (0.4)	0.599
My interest in trauma care has increased.	Post-training	4.4 (0.9)	4.6 (0.8)	0.423
	Post-evaluation	4.6 (0.6)	4.6 (0.5)	1.000
I am confident to perform hemostatic procedures for a cardiac injury.	Post-training	3.2 (1.0)	3.7 (0.7)	0.140
	Post-evaluation	3.7 (0.9)	3.8 (0.7)	0.770
I would recommend this training to my colleagues.	Post-training	4.3 (0.7)	4.6 (0.5)	0.318
	Post-evaluation	4.6 (0.6)	4.7 (0.5)	1.000
I obtained new knowledge and skills to achieve hemostasis of a cardiac injury.	Post-training	4.6 (0.6)	4.9 (0.4)	0.247
	Post-evaluation	4.6 (0.5)	4.8 (0.6)	0.202
I would like to repeat this training.	Post-training	4.3 (0.8)	4.5 (0.6)	0.495
	Post-evaluation	4.8 (0.4)	4.6 (0.5)	0.495

Scores shown are mean (Standard Deviation), using a 5-point Likert scale (range 1–5). *P*-values shown are comparing the two study groups, ex-vivo and live tissue

Table 7 shows the assessment of self-confidence comparing the results at all three time points, pre-training, post-training and post evaluation. Confidence levels are similar in the two study groups at the pre-training time point, with both groups showing significantly more confidence both post-training and post-evaluation. Comparing post-evaluation to post-training shows that the two study groups had similar confidence levels after the evaluation session, but that the live tissue group felt more confident at post-training.

**Table 7:** Confidence level comparing pre-training, post-training and post-evaluation

Study group (*n* = number of participants)	Pre-training	Post-training	Post-evaluation
	(*n* = 31)	(*n* = 31)	(*n* = 31)
Ex-vivo	1.7 (0.8)	3.2 (1.0), *p* = 0.00	3.7 (0.9), *p* = 0.00
Live tissue	1.9 (1.0)	3.7 (0.7), *p* = 0.00, p** < .05	3.8 (0.7), *p* = 0.00

Scores shown are mean (Standard Deviation), using a 5-point Likert scale (range 1–5). *P*-values shown are comparison with the pre-training questionnaire for each of the two study groups, while p** compares the live tissue and ex-vivo group for the post-training evaluation (Wilcoxon signed-rank test)

Interestingly, after the training session, the live tissue group had significantly more self-confidence (score 3.7) than the ex-vivo (score 3.2, p < .05) trained group. However, after the evaluation session, while the self-confidence of both groups was significantly greater compared to pre-training, there was no longer a difference in the self-confidence level of the two groups.

## Discussion

We conducted training using an ex-vivo model with a pump, and compared it with use of a live animal model to teach the management of penetrating cardiac injuries. After training, all participants were objectively evaluated in their management of a penetrating cardiac injury. All participants were able to achieve hemostasis at the injury site on the surface of the right ventricle. Objective assessment revealed that ex-vivo training was as effective as live tissue training, based on the evaluation conducted. The reliability and validity of the OSATS instrument were good. Participant responses to subjective questionnaires were positive and the level of self-efficacy increased.

Live tissue training is considered by some to be the most effective way to learn hemostatic skills for trauma surgery [6, 7]. Other training models, such as cadaver training, bench model training, or virtual reality training, do not have active blood circulation to mimic live tissue training. However, the ex-vivo system used in this study simulates active bleeding with an external pump which facilitates learning hemostatic procedures in a more realistic environment without a live animal [8].

Ethical issues regarding the use of experimental animals are also important [7] and ex-vivo training models are a good response to these ethical issues. Burch and Russel proposed the 3R’s for animal ethics [12], including Reduction, Replacement and Refinement. Although cadaver training and virtual reality training are beneficial from an ethical standpoint, these models may not be suitable for learning hemostatic skills. There are few studies comparing the effectiveness of training using these modalities with training using a live animal model [7]. The ex-vivo model described here using a pump provides the opportunity to learn hemostatic skills in trauma surgery with objective results similar to training with a live animal and respects the principles represented by the 3R’s.

Although we did not calculate exact costs in this study, estimates can be made, based on average charges at our institution. The direct cost of the ex-vivo training is the facility fee and the price of the sutures. The facility fee is equivalent to the electrical charges of the circulation pump and the refrigerator for preservation of the harvested organs, which we estimate to about US$20 for one session. The sutures cost about US$50 per session, for a total cost of about US$70. Live tissue training requires the cost of the pig, the fee for general anesthesia, and the sutures. The pig costs about US$1800, and the cost of anesthesia and the facility fee is about US$300 for a total cost of about US$2150, with the sutures. While these are only estimates, the cost of live tissue training is far greater than ex-vivo training.

One factor which influences the results of the objective evaluation is the size of the penetrating cardiac injury. The amount of hemorrhage was not excessive because the stab wound was just one centimeter long. It was relatively easy to stop the bleeding. If the injury were larger, hemostasis would be more difficult and a greater difference in results may have been observed. Another important factor is the location of the wound in the heart, which directly affects the ease of achieving hemostasis. If the injury was created in the left atrium, the results may have been different. Manipulation of an ex-vivo heart during the procedure does not have a physiologic effect, limiting the reality of the simulation.

The overall evaluation of the ex-vivo group was statistically significantly higher than that of the live tissue group. Among other evaluation items, such as amount of hemorrhage and time required to repair, there was no significant difference between the two groups, but the mean scores of the ex-vivo group are higher than those of the live tissue group, though the characteristics of participants are similar in both groups. The pressure of the bleeding with the ex-vivo model, seemed higher than that in the live tissue model according to some participants [8], and might influence the difference in scores. The impression of higher pressure bleeding with the ex-vivo model might give participants the impression that repair of the injury in the live tissue model is easier than during the training session.

The OSATS instrument had good reliability and validity and was useful as an evaluation tool. OSATS is widely used for the evaluation of technical skills in simulation [10, 11, 13–16]. The internal consistency reliability and inter-rater reliability analysis of the Global Rating Scale were high and those for the checklist were moderate. The validity was evaluated by comparing the results of residents and staff surgeons, and a significant difference was observed, which means that the OSATS used in this study can identify differences between the study groups. Overall, the evaluation tools demonstrated that training with this ex-vivo model is as useful as live tissue training for the management of a penetrating cardiac injury in a porcine model simple.

The subjective evaluations also revealed the usefulness of training with this ex-vivo model. All responses to items in the questionnaires showed that the training was perceived well by the participants. Self-confidence in both groups significantly increased after the training. Self-confidence carries the same meaning as self-efficacy, which was advocated by Bandura in 1977 [17]. Bandura described that self-efficacy means”people’s judgments of their capabilities to organize and execute courses of action required to attain designated types of performances [18].” In other words, it is a belief that one can accomplish a specific task. Self-efficacy is a very important characteristic for physicians and surgeons who perform procedures [19], particularly in emergency situations [20, 21]. Training with the ex-vivo model significantly increased the self-efficacy of participants for the repair of a penetrating cardiac injury. It was of interest that the increased self-efficacy in the live tissue trained group compared to the ex-vivo group was not sustained. Self-efficacy of the two groups was equal after the evaluation session.

This training is suitable for resident training as it facilitates repetitive practice. Aboud et al. reported that cadaver training for placement of an Intra-Aortic Balloon can be performed with a fluid pump to mimic circulation [22], but based on the description of the procedure, is far more complex than the system employed in this training program. This ex-vivo model is reasonable and needs less work to perform the training.

There are some limitations to this study. First, hemostasis is just one factor in the care of trauma patients. There are other technical skills required to save patients with penetrating cardiac injuries, such as performing a thoracotomy or median sternotomy to gain access to the injury site. Decision making and team dynamics for trauma are extremely important in emergency care. This ex-vivo training does not provide training for all of the requisite skills. Actual patient care experience in clinical practice is essential. There are a number of formal training programs to teach skills needed in the care of trauma patients. Second, the physical features of live tissue cannot be reproduced completely with this ex-vivo model, such as coagulation and motion of the heart. Third, a complex injury cannot be simulated using this ex-vivo model. Management of a single type of injury is a limitation using this model and this level of fidelity is suitable for learning basic procedures in trauma surgery. Fourth, the sample size is somewhat small. As Table 1 shows, there are differences between the two groups for time since graduation and prior experience (number of procedures) in chest and abdominal trauma surgery. While the differences are not significant, a significant difference may be identified with a larger sample size. Power analysis shows that the sample size required is 186 (α = 0.05, 1-β = 0.8), supporting the need for further study of the ex-vivo model.

Although this ex-vivo model has some limitations, simulation is an important modality in training for the care of injured patients. Utilizing this ex-vivo model is intended to bolster the competence of surgeons, and will hopefully lead to the increased survival of severely injured patients.

## Conclusions

Ex-vivo training with a pump was as useful as live tissue training to learn hemostatic techniques for a penetrating cardiac injury. This training increased the self-efficacy of participants, and objective scores of results showed no differences for training with an ex-vivo model or live tissue. This study supports the use of ex-vivo models for training in hemostasis, at lower cost and respecting the principle of 3R’s for animal research.

## Additional files

Additional file 1: Raw Data Ex vivo training objective for submission. (XLSX 31 kb) Additional file 2: Raw Data Ex vivo training subjective for submission. (XLSX 15 kb)
